# Optimization via game theoretic control

**DOI:** 10.1093/nsr/nwaa019

**Published:** 2020-03-19

**Authors:** Daizhan Cheng, Zequn Liu

**Affiliations:** Key Laboratory of Systems and Control, Academy of Mathematics and Systems Sciences, Chinese Academy of Sciences, China; Key Laboratory of Systems and Control, Academy of Mathematics and Systems Sciences, Chinese Academy of Sciences, China; School of Mathematical Sciences, University of Chinese Academy of Sciences, China

## Abstract

Using game theoretic control to solve optimization problem is a recently developed promising method. The key technique is to convert a networked system into a potential game, with a pre-assigned criterion as the potential function. An algorithm is designed for updating strategies to reach a Nash equilibrium (i.e. optimal solution).

## INTRODUCTION

Game-based control is a cross discipline of control theory and game theory. In a certain sense, control theory and game theory are twin brothers, born in the 1940s. They have a common task, that is, to manipulate certain objects to reach preassigned goals. However, one of the major difference is that the objects of controls are not intelligent, whereas the objects of games are intelligent, and are therefore subject to ‘anti-control’. Some authors claim that the control system is a particular kind of ‘games’. However, this demarcation line is murky. It was noted in Ref. [1] that when facing some intelligent objects such as intelligent machines and intelligent networks etc., the existing control theory may not be applied directly. Because the controllers and the controlled objects may have game-like interactions. Putting certain game factors into control framework is an important research topic, which is inevitable in dealing with some social and economical problems. Meanwhile, combining game factors can tremendously enlarge the development of control theory and its applications.

**Figure 1.**
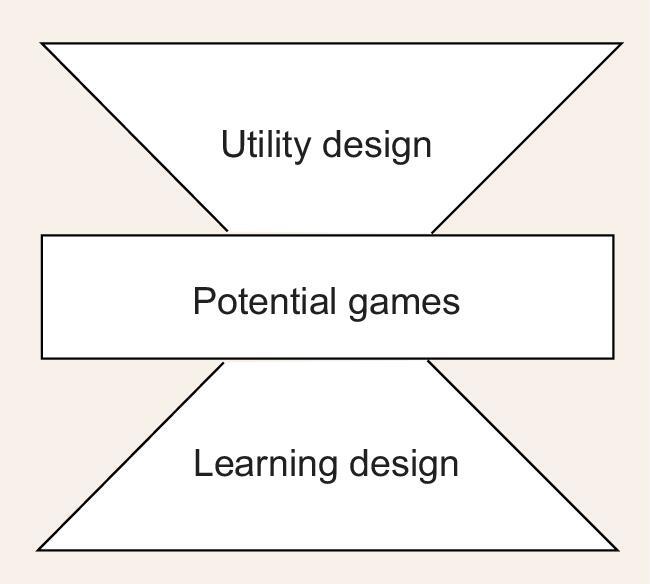
Game theoretic control.

There are many successful applications of game theory to control. For instance, the distributed coverage of graphs [2], which is important in sensor allocation, formation control of multi-agent systems, etc.; the congestion game based control, which is useful for transportation control, optimization of facility-based networks, etc.; and Nash equilibrium oriented control, which is applied to scheduling and resource allocation of networked process [3,4].

This perspective will focus on game theoretic control (GTC), which is particularly useful for optimization of networked systems. Gopalakrishnan *et al.* [5] describe a framework for optimization of multi-agent control problems using game theory. They propose an hourglass architecture to illustrate the GTC using potential games as the interface (Fig. 1). This approach is mainly based on potential game theory. There are some other useful game theory-based control design techniques. For example, Stackelberg game theory has been used to control agent behaviors [6,7].

Roughly speaking, optimization via GTC can be described as follows: consider a networked multi-agent system with *n* agents. Assume there is a global objective function *J*(*x*_1_, *x*_2_, …, *x*_*n*_), where *x*_*i*_ is the action (or strategy) of the *i*-th agent, and *J* could be a total cost, which will be minimized, or a total payment, which will be maximized. As shown in Fig. 1, the GTC approach consists of two major steps: (i) design utility (or payoff) functions for each agent such that the overall system becomes a potential game with *J* as its potential function; (ii) design a strategy updating rule (more precisely, a learning algorithm), such that when each agent is optimizing its own utility functions the system can converge to a Nash equilibrium, which is an optimal value of *J*. But it might be a local optimal value. Note that, in general, as each agent can only obtain its neighbor’s information, the learning algorithm must be based on local information. In the following sections we describe this in detail.

## POTENTIAL GAME

**Definition 2.1**

- A finite game is denoted by *G* = {*N*, *S*, *u*}, where (1) *N* = {1, $\ldots$, *n*} is the set of players; (2) $S=\prod _{i=1}^nS_i$ is the profile of strategies (or actions), where *S*_*i*_ = {1, $\ldots$, *k*_*i*_} is the set of strategies of player *i*; (3) *u* = (*u*_1_, $\ldots$, *u*_*n*_) and each $u_i:S\rightarrow {\mathbb {R}}$ is the utility (or payoff) function of player *i*.
- A finite game *G* = {*N*, *S*, *u*} is called a potential game, if there exists a function $P:S\rightarrow {\mathbb {R}}$, called the potential function, such that for every *i* ∈ *N* and for every *s*_−*i*_ ∈ *S*_−*i*_ ≔ $\prod $_*j* ≠ *i*_*S*_*j*_ and ∀α, β ∈ *S*_*i*_,

(1) \begin{eqnarray*} &&u_i(\alpha , s_{-i})-u_i(\beta ,s_{-i})\\ &&=P(\alpha , s_{-i})-P(\beta ,s_{-i}). \end{eqnarray*}

In a game-based optimization problem, the potential function plays a fundamental rule, which is similar to a Lyapunov function for stabilization of dynamic systems. The importance of potential function in GTC can also be seen from Fig. 1. Unfortunately, verifying whether a finite game is potential is not an easy job. This long standing problem was solved in Ref. [8], using a semi-tensor product (STP), which is defined as follows.

Definition 2.2. Let $A\in {\mathcal {M}}_{m\times n}$, $B\in {\mathcal {M}}_{p\times q}$, and $t=(n,p)$ be the least common multiple of *n* and *p*. Then the (left) STP of *A* and *B*, denoted by *A*⋉*B*, is defined as

(2) \begin{equation*} A\ltimes B:=\left(A\otimes I_{t/n}\right)\left(B\otimes I_{t/p}\right), \end{equation*}

where ⊗ is the Kronecker product.

As a convention, hereafter the default matrix product is STP, that is, *AB* = *A*⋉*B*.

To use STP, the action *a*_*i*_ ∈ *S*_*i*_ is denoted as $S_i=\lbrace \delta _{k_i}^j\,\,|\,\,j=1,\ldots ,k_i\rbrace$, where *j* ∈ *S*_*i*_ is expressed as $j\sim \delta _{k_i}^j$. Then each utility function *u*_*i*_ can be expressed as

(3) \begin{equation*} u_i=V^u_i\ltimes _{j=1}^na_j,\quad i=1,\ldots ,n, \end{equation*}

where $V^u_i\in {\mathbb {R}}^{k}$ is called the structure vector of *u*_*i*_ ($k:=\prod _{i=1}^nk_i$).

Construct a potential equation:

(4) \begin{equation*} E\!\xi = b, \end{equation*}

where

$$\begin{eqnarray*}
E&=&\left[{\begin{array}{ccccc}-E_1 &\quad\! E_2&\quad\! 0&\quad\! \ldots &\quad\!0\\
-E_1&\quad\!0&\quad\! E_3&\quad\!\ldots &\quad\!0\\
\vdots &\quad\! &\quad\!&\quad\!\ddots \\
-E_1&\quad\!0&\quad\!0&\quad\!\ldots &\quad\! E_n \end{array}}\right];\quad\nonumber\\ \xi &=&\left[{\begin{array}{c}\xi _1\\
\xi _2\\
\vdots \\
\xi _n \end{array}}\right];\quad b=\left[{\begin{array}{c}b_2\\
b_3\\
\vdots \\
b_n \end{array}}\right],
\end{eqnarray*}$$

and

$$\begin{eqnarray*}
E_i &=& I_{k^{i-1}}\otimes {\bf 1}_k\otimes I_{k^{n-i}}\nonumber\\
&&\in {\mathcal {M}}_{k^n\times k^{n-1}},\quad i=1,\ldots ,n.
\end{eqnarray*}$$

$$\begin{eqnarray*}
\xi _i:&=&\left(V^d_i\right)^T\in {\mathbb {R}}^{k^{n-1}},\quad\nonumber\\
&&i= 1,\ldots ,n.
\end{eqnarray*}$$

$$\begin{eqnarray*}
b_i:&=&\left(V^u_i-V^u_1\right)^T\in {\mathbb {R}}^{k^n},\quad\nonumber\\
&&i=2,\ldots ,n.
\end{eqnarray*}$$

Then we have the following result.

Theorem 2.3. A finite game  *G* is potential, if and only if, its potential Eq. (4) has a solution [8]. Moreover, if a solution exists, then the potential function has its structure vector as follows:

(5) \begin{eqnarray*} V_P&=& V^u_1-V^d_1M_1\nonumber\\ &=& V^u_1-\xi _1^T\left({\bf 1}_k^T\otimes I_k\right). \end{eqnarray*}

Using a potential equation, Liu and Zu [9] designed an efficient algorithm. Potential Eq. (4) can be easily extended to weighted potential games. Properly choosing weights can extend the application of potential game based optimization to a considerably larger set of systems. As the potential equation is a linear algebraic system, it is easy to figure out that the set of potential games is a vector subspace of finite games [10]. This vector space structure is very useful when near potential games are also used for optimization, because ‘near’ can be described by the topology of Euclidian vector spaces.

## UTILITY DESIGN

From Fig. 1, it can be seen that the utility design is one of the key issues in GTC. The utility design faces two major problems. First, as the global performance criterion *J* is given, one must know whether it is possible to design utility functions, that turn the overall system into a potential game with *J* as its potential function. Second, if this is possible, how can these be designed?

Take a facility based system as an example. When a facility based system is considered, a congestion game is a proper tool to model the system.

Definition 3.1. A facility based system is described by  $\Sigma =(M,N, ({\mathcal {A}}^i)_{i\in N}, P)$, where *M* = {1, 2, $\ldots$, *m*} is the set of  facilities, *N* = {1, 2, $\ldots$, *n*} are users and ${\mathcal {A}}^i\subset 2^M$ describes the strategies of user  *i*, which forms a set of subsets of *M*.  ${\mathcal {A}}:=\prod _{i=1}^n{\mathcal {A}}^i$ is a profile.  $P:{\mathcal {A}}\rightarrow {\mathbb {R}}$ is the total cost. The facility optimization problem is to find the best profile  $a^*\in {\mathcal {A}}$, which minimizes the cost. That is,

(6) \begin{equation*} P\!(a^*)=\min _{a\in {\mathcal {A}}}P\!(a). \end{equation*}

It is well known that a congestion game is a potential game. Using STP technique, Han *et al.* [11] obtained necessary and sufficient conditions for the solvability. Moreover, a design method is also proposed.

Let  $|{\mathcal {A}}|=\ell$, where  ${\mathcal {A}}=\lbrace A_1,A_2,\ldots ,A_{\ell }\rbrace$. Construct

(7) \begin{equation*} B\Xi ^T=P, \end{equation*}

where Ξ is the itemized cost, depending on the number of users,  $B:{\mathcal {A}}\rightarrow {\mathbb {R}}^n$ is the cost vector, and  *P* is the total cost.

Theorem 3.2 Given a facility based system  $\Sigma =(M,N, ({\mathcal {A}}^i)_{i\in N}, P)$, there exist cost functions such that the overall cost  *P*(*a*) becomes a potential function, if and only if, Eq. (7) has a solution [11].

The detailed design technique for cost functions is based on Ref. [11].

## LEARNING ALGORITHM

When a game is performed repeatedly, each agent is able to improve its strategies through learning. Then we have a dynamic game. Learning algorithm is also called the strategy updating rule. That is, how each agent updates its strategy to maximize (or minimize) its own utility function. When a strategy updating rule is decided, the dynamics of a dynamic game are also determined. Say, a Markov-type dynamic game is described as

(8) \begin{equation*} \left\lbrace \begin{array}{@{}l@{\quad }l@{}}x_1(t+1)=f_1(x_1(t),\ldots ,\\ \qquad x_n(t),u_1(t),\ldots ,u_m(t))\\ x_2(t+1)=f_2(x_1(t),\ldots ,\\ \qquad x_n\!(t),u_1(t),\ldots ,u_m(t))\\ \ldots \\ x_n\!(t+1)=f_n\!(x_1(t),\ldots ,\\ \qquad x_n\!(t),u_1(t),\ldots ,u_m(t)). \end{array}\right. \end{equation*}

where *x*_*i*_(*t*), *i* = 1, $\ldots$, *n* are the strategies of agent  *i* at moment  *t*, *u*_*j*_(*t*), *j* = 1, $\ldots$, *m* are extra controls. A learning algorithm has to be designed such that as agents optimize their own utility functions, the system will converge to a Nash equilibrium or even a global optimal value of the performance criterion *J*.

Using STP, Eq. (8) can be converted into its algebraic state space representation as

(9) \begin{equation*} x(t+1)=Lu(t)x(t), \end{equation*}

where $x(t)=\ltimes _{j=1}^nx_j\!(t)$,  $u(t)=\ltimes _{s=1}^mu_s(t)$, $L\in {\mathcal {L}}_{k\times k}$ is a logical matrix. Cheng *et al.* [12] provides a detailed method to formulate dynamic games. In addition, using algebraic form Eq. (9), many useful properties can be obtained [12].

For a potential evolutionary game, some algorithms can lead the profile to a Nash equilibrium, for example, myopic best response adjustment. For potential games, a Nash equilibrium is a local optimal profile. Unless the Nash equilibrium is unique, a Nash equilibrium is not enough to assure global optimization.

To assure a global optimal solution, some more powerful algorithms need to be developed. Note that when global optimization is investigated, mixed strategies are usually unavoidable. Then the algorithm becomes a state-dependent Markov chain [13].

## CONCLUSION

Game-based control is a promising new technique in control theory. In particular, when the system has certain intelligent properties or a complicated system with uncertainties, certain game-like interactions exist between controllers and controlled objects. As a successful application of game-based control, when the optimization of multi-agent systems is considered, GTC becomes a powerful tool. This perspective describes the framework of GTC. It consists mainly of two steps: (1) design utility functions, which turn the overall system into a potential game with the preassigned performance criterion into the potential function; (2) design a local information based learning algorithm, which assures that as each agent optimizes its own utility functions, the overall optimization can be reached. Compared with distributed optimization, this approach is much more convenient and with fewer restrictions.
